# Evaluation of an Assertive Management and Integrated Care Service for Frequent Emergency Department Attenders with Substance Use Disorders: The Impact Project: Evaluating an assertive management service for frequent ED attenders with substance use disorders

**DOI:** 10.5334/ijic.5343

**Published:** 2020-04-23

**Authors:** Nicholas Lintzeris, Rachel M. Deacon, Marian Shanahan, James Clarke, Stephanie MacFarlane, Stefanie Leung, Michelle Schulz, Anthony Jackson, Daniel Khamoudes, David E. A. Gordon, Apo Demirkol

**Affiliations:** 1 The Langton Centre, Drug and Alcohol Services, South Eastern Sydney Local Health District, Surry Hills NSW, AU; 2 Discipline of Addiction Medicine, Sydney Medical School, The University of Sydney, AU; 3 Drug Policy Modelling Program, National Drug and Alcohol Research Centre, UNSW Medicine, The University of New South Wales, Sydney NSW, AU; 4 Prince of Wales Hospital Emergency Department, South Eastern Sydney Local Health District, Barker Street, Randwick, NSW, AU; 5 School of Public Health and Community Medicine, The University of New South Wales, AU

**Keywords:** assertive care, frequent ED attenders, substance use disorder, integrated care

## Abstract

**Introduction:**

Frequent attenders to Emergency Departments (ED) often have contributing substance use disorders (SUD), but there are few evaluations of relevant interventions. We examine one such pilot assertive management service set in Sydney, Australia (IMPACT), aimed at reducing hospital presentations and costs, and improving client outcomes.

**Methods:**

IMPACT eligibility criteria included moderate-to-severe SUD and ED attendance on ≥5 occasions in the previous year. A pre-post intervention design examined clients’ presentations and outcomes 6 months before and after participation to a comparison group of eligible clients who did not engage.

**Results:**

Between 2014 and 2015, 34 clients engaged in IMPACT, with 12 in the comparison group. Clients demonstrated significant reductions in preventable (p < 0.05) and non-preventable (p < 0.01) ED presentations and costs, and in hospital admissions and costs (p < 0.01). IMPACT clients also reported a significant reduction in use days for primary substance (p < 0.01). The comparison group had a significant reduction (p < 0.05) in non-preventable visits only.

**Conclusions:**

Assertive management services can be effective in preventing hospital presentations and costs for frequent ED attenders with SUDs and improving client outcomes, representing an effective integrated health approach. The IMPACT service has since been refined and integrated into routine care across a number of hospitals in Sydney, Australia.

## Introduction

Studies indicate a high prevalence (up to 40%) of substance use disorders (SUD) in people presenting to emergency departments (EDs) [1,2,3,4,5]. A recent study of 1,615 attendees to eight Australian hospitals found 39% screened positive for problematic substance use – 32% requiring a brief intervention and 7% requiring more intensive treatment [6].

A particular client group of concern are those with SUDs who are frequent ED attenders. A systematic review indicated frequent ED users comprise 4.5%–8% of all ED clients but account for 21%–28% of visits, and that substance use appears to be a major contributing factor in urban settings [7]. In Australia, frequent ED users (from here defined as 5 or more ED presentations per year in line with other Australian studies [8,9,10]) were more likely to be admitted to hospital, more likely to have a psychiatric (including substance use) diagnosis and more likely to self-discharge while waiting for care than non-frequent users [8].

D&A and ED clinicians working in South Eastern Sydney Local Health District (SESLHD) identified a gap in the health system’s response to frequent ED attenders with SUDs. They often present intoxicated, in psychosocial crisis, or after-hours when D&A Consultation Liaison (CL) services are unavailable to co-ordinate community care. Hospital based services cannot easily address the broader health and welfare needs of these clients – rather they necessarily focus on immediate acute health problems, and have limited capacity to follow up clients. Furthermore, navigating the standard referral pathways from acute hospital to community based services can be problematic for these clients, who have difficulty negotiating the fragmented mix of primary care, D&A, mental health and other community services as a result of a range of social, financial, cultural, cognitive, substance use, mental and physical problems. As such, many re-present to ED with a similar set of unmet health and social problems, contributing to a high burden on acute services, poor engagement with community based services, and poor health outcomes.

Reported approaches developed internationally to target frequent ED attenders with SUD can be generally categorised as involving client care plans (a specific management plan implemented when the client attends the ED) or assertive case management strategies (involving an ongoing clinical interaction which extends beyond the hospital into the community). In the mental health arena, assertive case management models can be effective in reducing hospital utilisation in frequent hospital attenders [11].

However, there have been few evaluations of such models targeting substance use issues in frequent ED attenders. The introduction of co-ordinated care plans (without assertive case management) for the 50-most frequent attending clients to an ED in Texas [12] resulted in a modest 5 to 10% reduction in ED presentations and hospital. A pre-post evaluation of multidisciplinary co-ordinated ED care plans for 24 frequent attenders with SUDs in a Vancouver hospital demonstrated a more substantial (3-fold) reduction in ED presentations [13].

Evaluations of assertive care management programs for this client group are more encouraging. An assertive management service in the UK resulted in a 67% reduction in the number of hospital admissions, and a 59% reduction in ED presentations in the three months following the intervention period [14]. Similar reductions in ED presentations were demonstrated in US programs [15,16]. Two Australian interventions included frequent ED attendees with and without SUDs. Phillips et al. [10] noted that although case management intervention did not reduce ED visits for their clients but increased the level of engagement with community based healthcare services. Grimmer Somers et al.’s [17] study demonstrated reduced ED presentations, emergency admissions, reduced hospital costs, increase in planned outpatient visits, primary care linkage and housing stability in the whole frequent ED attender patient group and the SUD sub-group (comprising half of the sample).

In summary, there is recognition of the importance of SUDs, often with co-morbid health and social problems, in frequent ED attenders, and the need for better co-ordination between acute and community care services. Whilst such programs may make intuitive or clinical ‘sense’, there have been few evaluations of integrated care models targeting this population, with existing studies comprising small sample sizes and often without control groups. No service models or programs focussing exclusively on people with SUDS have been reported in the Australian context.

In order to better address the needs of this client population, SESLHD D&A Services, in collaboration with ED and other stakeholders with funding through a SESLHD Innovation in Integrated Care grant program, established the IMPACT (**I**ntegrated **M**anagement **P**athways for **A**lcohol & drug **C**lients into **T**reatment) project – an assertive care management program targeting frequent attenders with SUDs at EDs within two SESLHD hospitals. The aims were to help address barriers interfering with clients’ ability to engage effectively with treatment (e.g. cognitive, mental health, physical health and social issues), enhance their participation in community based D&A, health and welfare services, improve their substance use, general health and welfare over time, and to reduce preventable hospital presentations (ED & admissions).

This paper describes the findings of an evaluation of the first 15-month intake of clients onto the program. The primary endpoints of the evaluation were to:

(1) *Describe characteristics of IMPACT clients*,
(2) *Measure IMPACT service costs, and*
(3) *Measure changes in hospital presentations and costs for IMPACT clients*.

The secondary endpoint was to

(4) *Measure* c*hanges in IMPACT client substance use, health and welfare outcomes*.

## Methods

### Design

A pre-post intervention study design examined a range of health service utilisation, health service costs, and clinical outcomes of clients of the IMPACT service. The IMPACT project commenced service delivery on 1 September 2014, and recruitment to this evaluation ceased on 19 December 2015. Data from two groups of clients are included in the evaluation: those assessed as eligible (meeting criteria) who participated in the IMPACT service, and a comparison group of clients assessed as eligible but who did not engage in the IMPACT service beyond their initial assessment. Although the IMPACT service was SESLHD-based, hospital presentation data was also obtained from a third nearby non-SESLHD hospital, which some IMPACT clients attended in addition to the SESLHD hospitals. The evaluation was approved by the SESLHD Human Research and Ethics Committee (no. LNR/14/POWH/483) including a waiver of consent for use and disclosure of medical information between the approved sites.

### Eligibility criteria

Eligibility criteria for the service were:

1. Attended local ED on 5 or more occasions in the previous 12 months;
2. A moderate to severe SUD (DSM-V criteria) contributing to ED presentations, and/or preventing the client from addressing important health or social issues;
3. Client not already engaged in another care co-ordination program;
4. Age 18 years or more; and
5. Voluntary verbal agreement from client to participate in the IMPACT service.

### Description of intervention: The IMPACT service

The IMPACT team comprised two social workers (each full time equivalent) and an Addiction Medicine specialist (0.1 full time equivalent) working in collaboration with the CL team. The social workers were primarily responsible for case management and coordination. The Addiction Medicine specialist as lead clinician provided oversight of client’s care.

Referrals were made by SESLHD ED clinicians to the D&A CL service who directed referrals to the IMPACT team. After screening, potentially eligible clients were followed up for assessment either in hospital or in the community (including home visits where needed). Eligible and consenting clients enrolled in the IMPACT service were offered assertive management services, which included

- a multidisciplinary assessment of client needs;
- development and implementation of a care plan in collaboration with the client and other service providers;
- facilitated referrals to relevant health (D&A, mental health, primary care, other specialist health) and welfare (housing, financial, legal and disability) support services, including support to book appointments and complete forms, and travel assistance;
- a hospital ED management plan for each client developed in collaboration with ED clinicians, with ‘alerts’ on the client’s electronic health records; and
- access to client transport assistance and brokerage funding to assist with essential one-off expenses.

Whilst there was no strict upper time limit for engagement with the IMPACT service, in general assertive care management lasted up to 6 months, enabling a case load of approximately 10 to 15 IMPACT clients per social worker at any one time. The IMPACT team met weekly for case review, and routinely engaged with other service providers as required.

On completion, care was transferred to an agreed provider, usually to the SESLHD D&A service. At times clients were linked into another D&A services (e.g. residential rehab), and/or referred onto another service (e.g. mental health or primary care).

### Measures and Outcomes

#### Characteristics of IMPACT clients

Demographics (age, gender, Aboriginal and/or Torres Strait Islander identity, principal substance of concern, homelessness and employment) were collected via clients’ initial clinical assessments.

#### IMPACT service costs

Costs of the IMPACT service included clinician time (both direct and indirect), clinician travel cost for community visits, and a brokerage fund. Clinician time was estimated using actual clinician hours spent on direct and indirect client contact. Other indirect clinician time included case management meetings (one hour per week for both social workers) and oversight by a staff specialist (0.1 fulltime equivalent). Time was costed using relevant 2015–2016 NSW Public Service salaries [18] plus superannuation and infrastructure costs; precise values used are available in Appendix table ii. Costs of local car travel for IMPACT workers were estimated by using the Australian Tax Office car expense deductions rate (per kilometre method) for the 2015/2016 financial year ($0.66/km) [19]. Travel was estimated from offsite appointments for one worker, multiplied by 1.4 to allow for the same amount of travel by the second worker and assuming 60% of all trips were taken by both workers. Brokerage costs were actual use per client.

#### Hospital presentations and costs

Hospital electronic records were extracted for IMPACT and comparison clients in the 6-month period prior to clients’ referral to the service, the period of IMPACT service delivery, and for 6 months after cessation of the IMPACT service for each client (or 6 months after assessment for comparison clients). The medical records of SESLHD ED presentations (non-SESLHD medical records were not able to be accessed by SESLHD clinicians) were jointly reviewed by an Addiction Medicine specialist (author AD) and two Emergency Physicians (authors DK and DEAG), and each presentation was assigned as ‘preventable’ or ‘non-preventable’. Preventable ED presentations were defined as those that may have been avoided with high quality primary and preventive care – if clinicians effectively diagnosed, treated, and educated clients, and if clients actively participated in their care and adopted healthy lifestyle behaviours [20]. Hospital admissions were classified as substance use related (ICD-10 principal diagnosis codes F10 to F19, and T40 to T43) or non-substance use related (other) [21] according to principal diagnosis on the discharge summary. Hospital ED and admission data was available for all 46 IMPACT and comparison clients.

Emergency department visit costs were estimated by using the average cost of ED presentations for all Urgency Related Groups based on the Australian Public Hospitals Cost Report 2013–2014 Round 18, stratified by triage code (1–5) and admission status [22]. Costs were adjusted to financial year 2015–2016 using Consumer Price Index changes for health between December 2013 and December 2015, calculated using the mid-points of 2013–2014 and 2015–2016 financial years based on Australian Bureau of Statistics data [23]. See Appendix table i for precise applied costs. Hospital admissions costs were calculated using assigned Diagnostic Related Groups codes, length of stay and clients’ Aboriginal identity (assuming psychiatric care days and leave days equal to 0 for all clients, acute care type, zero ICU hours for all visits, source of funding health service budget not covered elsewhere), using the Acute Admitted Services National Weighted Activity Unit calculator and a National Efficient Price of AUD 4,971 per National Weighted Activity Unit for the 2015–2016 financial year [24,25]. All costs are in Australian Dollars.

#### Substance use, health and welfare outcomes

Data was only available for those clients participating in the IMPACT service, not for those in the comparison group. Outcomes measures were:

- The Clinician Global Impression–Improvement Scale, a 7-point global scale of improvement in a client’s condition (1 = very much improved since initiation, 2 = much improved, 3 = minimally improved, 4 = no change, 5 = minimally worse, 6 = much worse, 7 = very much worse) [26]. A consensus rating was given by the IMPACT clinicians on completion of the IMPACT service for the separate domains of substance use, physical health, mental health and quality of life, as well as overall clinical change. Client outcomes were grouped as ‘improved’ for scores of 1–3, ‘no change’ for scores of 4, or ‘deteriorated’ for scores of 5–7.
- Client reported outcomes using the Australian Treatment Outcomes Profile, a validated 19-item clinical monitoring tool that assesses outcomes in the preceding 4 weeks [27]. The ATOP was collected at admission to the IMPACT service and over the course of treatment by the IMPACT clinicians. Health information including ATOPs were also collected independently by a researcher. Whilst the intention was for a researcher to follow up clients and conduct research interviews with them after their discharge from the IMPACT service, poor follow-up rates (less than 40%) resulted in too small a sample to reliably describe changes over time. Nevertheless, utilising both IMPACT clinician-completed and research-completed ATOPs furnished outcomes data for a majority of the sample. Where more than one ATOP was collected during the treatment episode, the last completed ATOP was used.

### Statistical analysis

Descriptive statistics were used to characterise the sample and compare preventable, non-preventable and total ED presentations and substance use related, non-substance use related and total hospital admissions before, during and after client involvement in the IMPACT service. Non-parametric tests were used to compare changes over time and between groups. Parametric tests were used to compare changes for ATOP data items.

Several clients of the IMPACT service had multiple interactions (i.e., they disengaged from the service, and re-engaged at a later time) which were aggregated into one episode per client. For the 4 deceased participants, ED and hospital visit rates post-IMPACT were not calculated.

## Results

### Characteristics of IMPACT clients

**Figure 1:**
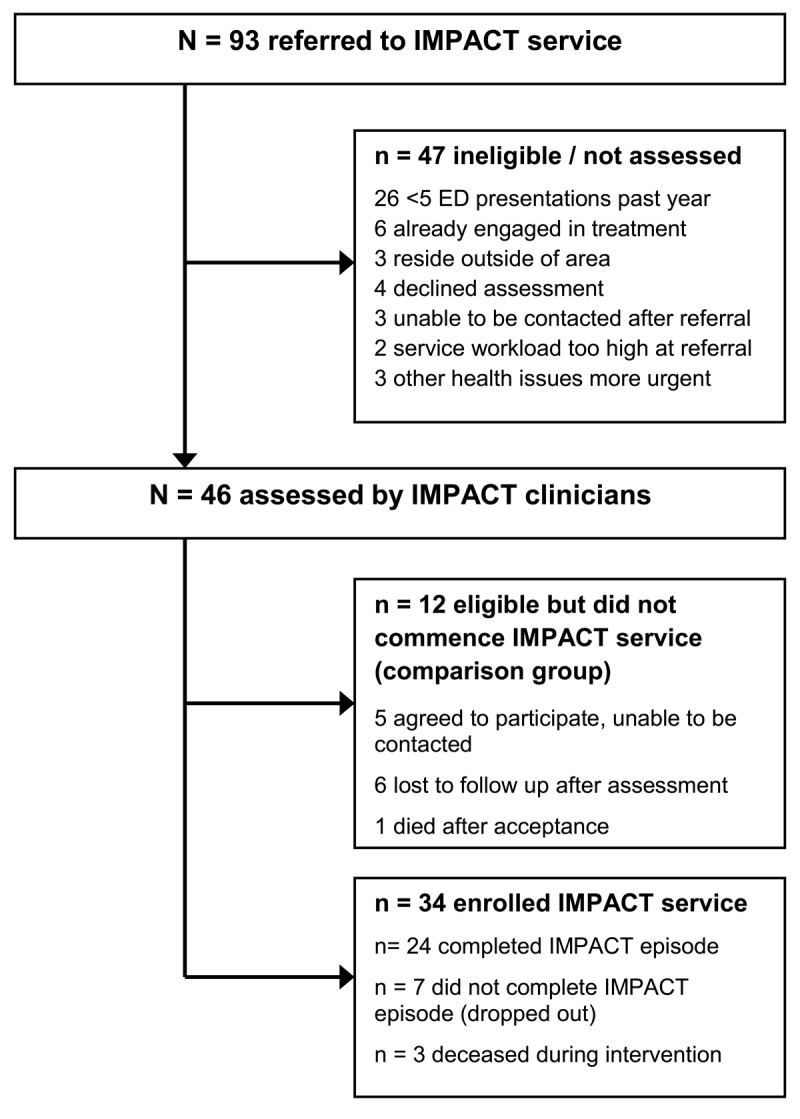
Participant flow diagram for the IMPACT service.

During the 15 month recruitment period, 93 clients were referred to the IMPACT service, of which 46 were found to be eligible by IMPACT clinicians and either enrolled in the IMPACT service (n = 34) or were assessed as eligible but did not commence an IMPACT service episode (the Comparison group, n = 12) (Figure 1).

The majority of IMPACT participants were female (n = 19, 56%), with a mean age of 45 years (standard deviation (SD) = 12). Primary substance of concern was alcohol (n = 22, 65%), polysubstance (n = 6, 18%) or stimulants, opioids or cannabis (n = 2, 6% each). Four clients (12%) identified as Aboriginal and/or Torres Strait Islander, n = 7 (21%) reported any recent paid employment, and n = 8 (23%) were currently homeless. The comparison group had similar characteristics, except that the majority (75%) were male (Fisher’s exact test, p = 0.096).

### IMPACT service costs

The mean duration of participation in the IMPACT service was 6.3 months (SD = 3.1; median 6.2; interquartile range 4.3–8.4 months). The mean number of occasions of service by an IMPACT clinician – including direct and indirect client contact – in each episode was n = 45 (SD = 33), and the median was n = 37.5 (interquartile range 17–68), equivalent to approximately 1.7 occasions of service per client per week. The mean costs per client episode were $2,213; comprising $1,214 (SD = $1,287) for occasions of service, $844 for clinical supervision, $221 for case management meetings, $35 (SD = $63) for brokerage, and $78 for transport (SDs were unavailable for items estimated from whole of service usage: supervision, case management meetings, and transport).

Twenty four (71%) clients were adjudged by IMPACT clinicians as successfully completing the IMPACT treatment episode (defined as discharge, or transfer to another service for ongoing care management), 7 clients failed to successfully complete (discontinued) the episode, and 4 clients died (3 in the IMPACT group and 1 in the comparison group), a crude mortality rate of 69.6 per 1000 person years with a 95% confidence interval 1.7–172.2 per 1000 person years. Each of these deaths was a result of an acute on chronic illness, with no immediate substance use cause identified.

### Hospital presentations and costs

Hospital utilisation data (ED presentations and admissions) are shown in Table 1 and related costs in Table 2. In the 6-month period prior to referral to the IMPACT service, clients were presenting to ED at a mean frequency of 1.4 times per month (median 1.0) in the IMPACT group, with the majority (64%) preventable presentations. There was a significant reduction (50% reduction) in total ED presentations – reducing to 0.7 in the 6-months after the intervention (p < 0.01). Significant reductions occurred in both preventable (56%; p = 0.013) and non-preventable (67%; p = 0.009) ED presentations. In contrast, clients in the Comparison group had a similar number of presentations in the 6-months prior to their referral (mean = 1.9, p = 0.57 compared to IMPACT clients), and whilst there was an overall significant reduction in mean non-preventable ED presentations to 0.1 per month (75% reduction; p = 0.010) in the 6 months after assessment, reductions in preventable and total ED presentations did not reach statistical significance.

**Table 1:** Comparison of ED attendances and hospital admissions per month among IMPACT and comparison clients in the 6 months prior to, during (IMPACT group only), and in the 6 months post-IMPACT involvement. ED attendances are categorised as either preventable or non-preventable (ED attendances at the non-SESLHD hospital were unable to be reviewed and are presented as unassigned), and admissions are classified as substance use-related or not.

ED attendances per month	IMPACT Group (n = 34)				Comparison Group (n = 12)		

	6-months prior	During IMPACT	6-months post IMPACT	Comparison of prior and post*	6-months prior	6-months post-assessment	Comparison of prior and post*

	mean (SD) median (95% CI)			Z, p	mean (SD) median (95% CI)		Z, p

*Preventable*	0.9 (0.9)	0.7 (0.7)	0.4 (0.7)	–	0.7 (0.8)	0.5 (1.0)	–
	0.5 (0.3, 1.2)	0.5 (0.3, 0.8)	0.2 (0.0, 0.3)	–2.493, 0.013	0.3 (0.0, 1.3)	0.2 (0.0, 0.5)	–0.638, 0.523
*Non-preventable*	0.3 (0.4)	0.2 (0.4)	0.1 (0.6)	–	0.4 (0.4)	0.1 (0.1)	–
	0.2 (0.0, 0.3)	0.1 (0.0, 0.2)	0.0 (0.0, 0.0)	–2.613, 0.009	0.4 (0.2, 0.7)	0.0 (0.0, 0.2)	–2.582, 0.010
*Unassigned*	0.2 (0.6)	0.5 (1.0)	0.1 (0.3)	–	0.8 (1.4)	0.5 (0.6)	–
	0.0 (0.0, 0.0)	0.0 (0.0, 0.3)	0.0 (0.0, 0.0)	–0.827, 0.408	0.1 (0.0, 1.3)	0.2 (0.0, 1.0)	–0.339, 0.735
*All*	1.4 (1.2)	1.4 (1.4);	0.7 (0.9);	–	1.9 (2.0)	1.1 (1.3)	–
	1.0 (0.7, 1.7)	1.2 (0.6, 1.4)	0.2 (0.0, 0.7)	–2.868, 0.004	1.2 (0.5, 2.0)	0.7 (0.2, 1.8)	–1.930, 0.054
**Hospital admissions per month**	**mean (SD) median (95% CI)**			**Z, p**	**mean (SD) median (95% CI)**		**Z, p**

*Substance use related*	0.5 (0.4)	0.4 (0.5)	0.2 (0.3)	–	0.2 (0.3)	0.1 (0.2)	–
	0.3 (0.2, 0.5)	0.2 (0.0, 0.5)	0.0 (0.0, 0.2)	–3.008, 0.003	0.0 (0.0, 0.3)	0.0 (0.0, 0.2)	–0.595, 0.552
*Unrelated to substance use*	0.3 (0.4)	0.3 (0.4)	0.1 (0.3)	–	0.2 (0.3)	0.2 (0.5)	–
	0.2 (0.0, 0.3)	0.2 (0.1, 0.3)	0.0 (0.0, 0.2)	–2.043, 0.041	0.2 (0.0, 0.3)	0.0 (0.0, 0.2)	–1.317, 0.188
*All*	0.8 (0.5)	0.7 (0.6)	0.3 (0.5)	–	0.4 (0.5)	0.3 (0.5)	–
	0.7 (0.5, 1.0)	0.7 (0.3, 0.9)	0.2 (0.0, 0.3)	–3.467, 0.001	0.3 (0.2, 0.5)	0.0 (0.0, 0.3)	–1.299, 0.194

* Wilcoxon signed-rank test.

**Table 2:** Comparison of Emergency Department (ED) attendance and hospital admissions total costs in Australian Dollars (financial year 2015–2016 equivalent) among IMPACT and comparison clients, in the 6 months prior to, during (IMPACT group only), and in the 6 months post-IMPACT involvement. ED attendances are categorised as either preventable or non-preventable (ED attendances at the non-SESLHD hospital were unable to be reviewed and are presented as unassigned), and admissions are classified as substance use related or not.

ED attendances total cost	IMPACT Group (n = 34)				Comparison Group (n = 12)		

	6-months prior	During IMPACT	6-months post IMPACT	Comparison of prior and post*	6-months prior	6-months post-assessment	Comparison of prior and post*

	mean (SD) median (95% CI)			Z, p	mean (SD) median (95% CI)		Z, p

*Preventable*	3,598 (3,397)	3,224 (4,174)	1,858 (2,853)	–	2,181 (2,430)	2,060 (3,409)	–
	2,647 (1,370, 3,769)	1,868 (1,035, 3,659)	630 (0, 2,070)	–2.687, 0.007	1,035 (0, 5,068)	459 (0, 3,105)	–0.059, 0.953
*Non-preventable*	1,561 (2,346)	1,246 (2,144)	883 (3,552)	–	1,968 (1,635)	373 (493)	–
	1,035 (0, 1,479)	630 (0, 1,035)	0 (0, 0)	–2.339, 0.019	1,633 (918, 3,046)	0 (0, 815)	–2.667, 0.008
*Unassigned*	1,122 (2,806)	1,888 (3,427)	392 (966)	–	2,592 (4,539)	999 (1,237)	–
	0 (0, 0)	0 (0, 1,443)	0 (0, 0)	–1.070, 0.285	204 (0, 5,393)	408 (0, 2,226)	–1.352, 0.176
*All*	6,281 (4,577)	6,359 (6,066)	3,133 (4,415)	–	6,741 (5,309)	3,432 (4,478)	–
	5,220 (3,166, 8,074)	3,746 (2,760, 8,510)	1,035 (0, 3,808)	2.998, 0.003	4,751 (3,282, 7,734)	1,490 (630, 3,215)	–2.118; 0.034
**Hospital admissions total cost**	**mean (SD) median (95% CI)**			**Z, p**	**mean (SD) median (95% CI)**		**Z, p**

*Substance use related*	11,720 (12,974)	11,988 (17,111)	8,066 (28,129)	–	4,120 (6,809)	1,688 (3,536)	–
	9,870 (4,644, 14,515)	6,685 (0, 11,684)	0 (0, 602)	–2.629, 0.009	0 (0, 7,537)	0 (0, 2,021)	–1.041, 0.310
*Unrelated to substance use*	13,631 (23,332)	15,679 (28,452)	6,842 (15,256)	–	5,927 (7,466)	884 (1,919)	–
	782 (0, 11,592)	5,621 (609, 10,479)	0 (0, 2,027)	–1.380, 0.168	4,971 (0, 9,622)	0 (0, 667)	–2.111, 0.035
*All*	25,351 (23,496)	27,667 (35,717)	14,908 (40,737)	–	10,047 (9,443)	2,572 (3,629)	–
	15,935 (12,106, 26,736)	14,641 (7,352, 25,077)	602 (0, 9,201)	–3.096, 0.002	6,273 (4,971, 13,113)	0 (0, 4,971)	–2.189, 0.029

* Wilcoxon signed-rank test.

Similar patterns were seen in hospital admissions – a 62% reduction in total admissions, with significant reductions in both substance use (60%) and non-substance use related (67%) admissions in the IMPACT group. There was no statistically significant reduction in hospital admissions in the Comparison group.

Despite marked individual variation, there were significant reductions in total ED and inpatient admission hospital costs in both groups over time. IMPACT clients had significant reductions in ED costs for both preventable and non-preventable presentations, whereas the Comparison clients only had significant reductions in non-preventable ED presentations. Significant reductions were seen for substance use related inpatient admission costs in the IMPACT group, whereas for the Comparison group, significant reductions were only seen for the admission costs unrelated to substance use.

### Substance use, health and welfare outcomes

Client reported ATOP outcomes comparing baseline and end of their participation in the IMPACT service for 23/34 (68%) of clients are shown in Table 3. The mean time between baseline and follow-up ATOPs was 170 days. There were statistically significant reductions in days of primary substance use (t(degrees of freedom) = 3.316 (22), p < 0.01), and improvements in Quality of Life score (t(degrees of freedom) = –2.085 (16), p = 0.054).

**Table 3:** Baseline and follow up client-reported outcomes for the 23/34 IMPACT clients with an Australian Treatment Outcomes Profile completed.

ATOP item	Number of clients with ATOP item available	Baseline	Follow-up	p

*Days primary substance use in previous 28, mean (95% CI)*	23	19 (15, 22)	11 (6, 15)	0.003*
*Psychological health, self-rated on a 0 (poor)-10 (good) scale, mean (95% CI)*	17	5.2 (4.1, 6.4)	6.1 (4.7, 7.6)	0.209*
*Physical health, mean (95% CI)*	16	4.6 (3.3, 5.9)	5.7 (4.4, 7.0)	0.142*
*Quality of life, mean (95% CI)*	17	4.6 (3.4, 5.9)	6.3 (4.7, 7.9)	0.054*
*Homeless/at risk in previous 28 days, % (95% CI)*	22	26% (11%, 50%)	35% (17%, 59%)	0.688**
*Been arrested, % (95% CI)*	23	13% (3%, 34%)	13% (3%, 34%)	1.000**
*Any violence, % (95% CI)*	23	43% (23%, 65%)	22% (7%, 44%)	0.180**
*Any employment/education, % (95% CI)*	23	17% (5%, 39%)	13% (3%, 34%)	1.000**

* Paired t-test. ** McNemar’s test and binomial test with Copper-Pearson exact confidence intervals.

**Figure 2:**
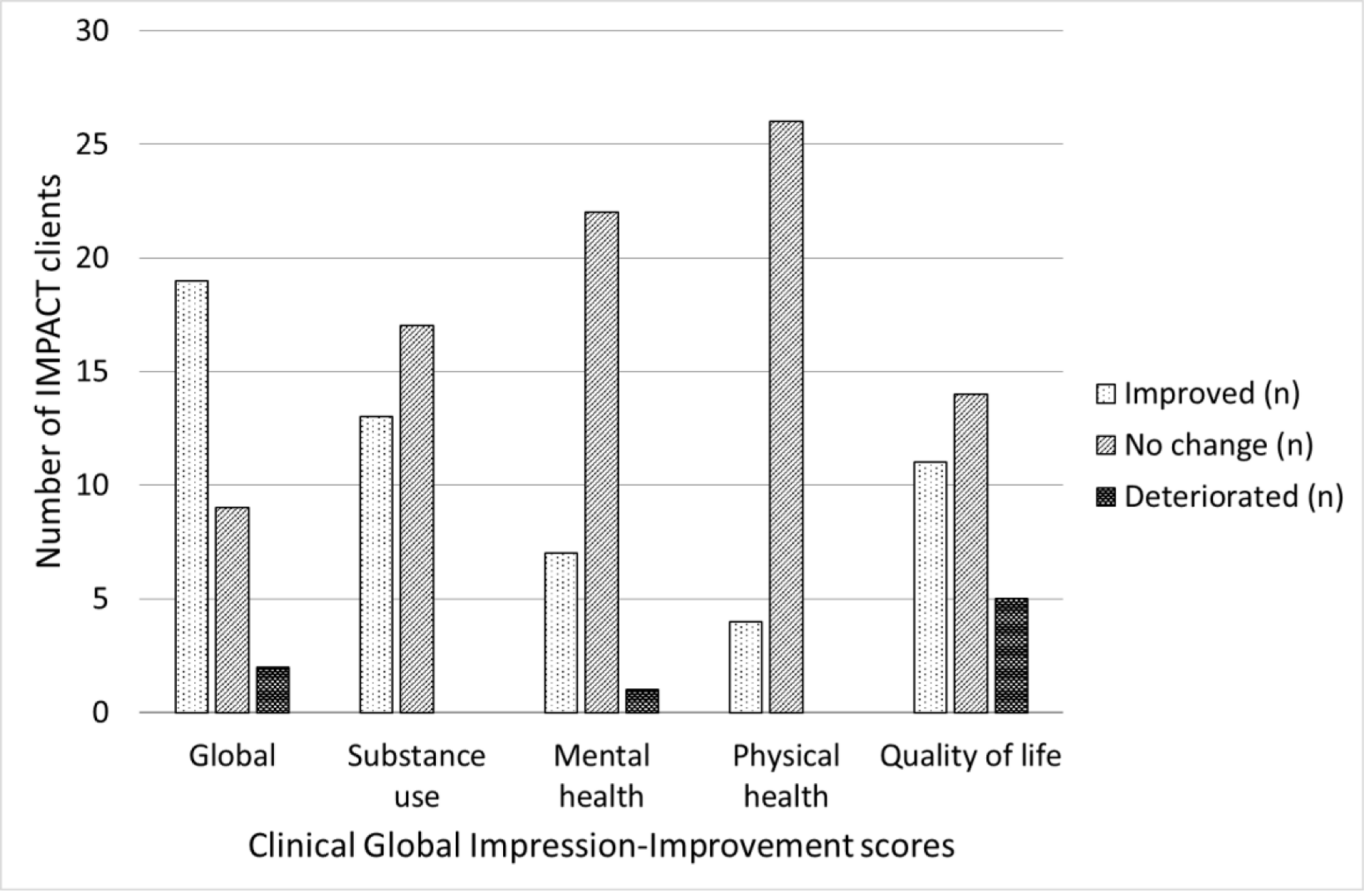
Clinical Global Impression-Improvement scores for 30 out of 34 clients of the IMPACT service (excluding 3 deceased clients and 1 unable to be rated), a 7-point scale whereby treating clinicians globally rate the level of improvement in the client condition over the course of the treatment episode (1 = very much improved since initiation, 2 = much improved, 3 = minimally improved, 4 = no change, 5 = minimally worse, 6 = much worse, 7 = very much worse). Clients were scored on global health, substance use, mental health, physical health and quality of life domains. Client outcomes were considered ‘improved’ for scores of 1–3, ‘no change’ for scores of 4, or ‘deteriorated’ for scores of 5–7.

Clinician ratings for IMPACT participants using the CGI-I are shown in Figure 2. Clinicians rated that just over half the IMPACT clients (58%) generally improved their overall condition over the course of the IMPACT episode, although a minority (15%) deteriorated. Consistent with client reported outcomes, improvements were mostly seen in the areas of substance use and quality of life, whereas improvements in physical and mental health domains across the group were less pronounced. Global improvements were associated with a decrease in ED visit rate (p = 0.009, Wilcoxon signed-rank test), whereas there was no change in ED visit rate for clients with no change or who deteriorated.

## Discussion

We report on the first evaluation of an assertive management approach for frequent ED attenders with moderate to severe SUDs in an urban Australian setting. The study examined the effects of the service upon hospital presentations, service costs and client outcomes, with a comparison group of similar clients. The IMPACT service operated within a broader specialist D&A treatment service, with close links to hospital services (ED, hospital D&A CL, mental health), and primary health and welfare services in the community. The model represents an integrated care approach to this client group with complex service needs.

For the clients enrolled in the IMPACT service most ED presentations identified as ‘preventable’; and most inpatient admissions were for a substance use related primary diagnosis. Alcohol was the main primary substance of concern, although polysubstance use was common, and clients had a range of mental, physical and social problems, including low employment, homelessness and experience of violence. That there were more women in the active group may have been a function of the proactive and trauma-informed approach employed, enabling women who were referred to IMPACT to engage better than with D&A services without outreach. Previous work indicates women face greater barriers to seeking help from D&A treatment services than men [28].

The severity of the health concerns for this population is highlighted by the high crude mortality rate. Although this figure needs to be treated with caution given the small sample size, there being four deceased clients out of 46 during the 15 month evaluation period is compelling.

Consistent with previous evaluations, a multidisciplinary health and social welfare approach based on an assertive management model that spanned community and hospital settings was effective in achieving the service goals. There were significant reductions in hospitalisations, most notably in preventable ED presentations and substance use related inpatient admissions. There was an overall reduction in ED presentations by approximately 50% in the 6-months before and after the service, and a 63% reduction in hospital admissions.

Whilst reductions in ED presentations and hospital admissions were also seen in the Comparison group, these were less pronounced than in the intervention group, and were not statistically significant regarding preventable ED presentations or substance use-related hospital admissions. The ‘natural’ reduction (without any specific intervention) in ED presentations in frequent ED attenders has been previously reported in several studies (see [5]), but importantly, the changes were more pronounced for IMPACT clients compared to the control group.

The direct costs of delivering IMPACT were approximately $AUD2,213 per episode, with the mean duration per episode of 6 months. The reductions in hospital ED and inpatient admission costs were considerably greater, suggesting potential overall savings to the health system with the use of such assertive care models.

Whereas previous evaluations have generally reported only on hospital outcomes, we were also able to demonstrate significant improvements in both client and clinician reported domains of substance use and overall quality of life, with improvements in mental and physical health domains. It should be noted that not all clients benefited from the service, highlighting the need for further service improvements, and the complexity of problems faced by this client group.

The study has some significant limitations. The low participant numbers suggest caution is required in generalising conclusions. Nevertheless, the study has higher or similar numbers than in previous evaluations of similar programs, and the general findings are consistent with previous reports [14,15,16]. While our study had a comparison group, these participants were not randomly allocated. However, the ethical difficulties of random assignment to a ‘control’ group for these clients are considerable. Finally, although the study design originally aimed to have independent researchers following up and interviewing participants to examine client reported outcomes and broader health service utilisation, we achieved low research follow up rates (less than 40%), highlighting the difficulties of engaging this client group in direct-contact research. Previous evaluations also note difficulties in following up clients [10,17]. Nevertheless, the importance of measuring client-reported outcomes for substance use, health and welfare as well as service-reported outcomes remains, and we encourage future studies to capture these data where possible.

Only data from three geographically close hospitals were included. It is likely that some clients occasionally sought healthcare from other hospitals located further away, but these data was not sought due to resource limitations. Also unknown is the extent to which clients changed their healthcare seeking patterns due to their awareness about being identified as frequent attenders. An unintended consequence of such a reduction in presentations may have been a poorer health outcome for some clients. However, the association between improved global CGI-I scores and reduced ED visits does not support this conclusion as a whole.

### Implementation of the model of care following the IMPACT pilot project

IMPACT was the first assertive community service in New South Wales targeting patients with severe SUDs and frequent ED presentations. The demonstration of the feasibility of the clinical model of care, and the positive outcomes for both services and patients, led to the refinement and expansion of the model of care across several regions in NSW, and renamed the AoD Assertive Community Management (ACM) teams. At the time of writing there are 5 ACM teams funded by NSW Health and operational across metropolitan and regional NSW. The key refinements were:

- *Broadening of referral pathways*: The new ACM service no longer require patients to meet strict ED presentation criteria, and now accepts referrals from a broader range of health service providers, targeting patients with severe SUD and a history of poor engagement with AoD services. This recognises the somewhat arbitrary ‘cut-off’ of 5 presentation to one ED under IMPACT, and that in many cases, referral to the ACM may prevent a spate of ED and hospital presentations.
- *Broader multidisciplinary composition of ACM teams*: The original IMPACT service was largely staffed by social workers, with some medical and nursing input. The experience of implementing IMPACT highlighted the very high proportion of patients with significant cognitive impairment; and the need for better assessment of the functional needs of patients regarding accommodation, managing finances and other skills required for independent living in the community. To this end, the ACM services prioritise the need for part-time neuropsychologist and occupational therapists, with the ability for home visiting and assessments of functioning in the community.
- *‘Regional’ networking of ACM services*. The first IMPACT service was a feasibility project implemented in one metropolitan hospital. However, particularly in urban areas such as Sydney (population over 5.5M), it is recognised that patients often attend a number of hospitals, particularly in high density inner city areas where several hospitals are in close geographic proximity, and also that a considerable proportion of these patients have unstable housing such that hospital ‘catchment areas’ are often meaningless for the patient. The implementation of ACM has resulted in three of the five ACM teams (SESLHD, St Vincent’s Hospital Network and Sydney Local Health District, representing 7 hospitals with a population catchment of approximately 1.7M) working closely together with similar operating procedures, shared electronic medical records, regular communication, case conferencing and weekly meetings between ACM teams.
- *Further refinement of funding models of ACM teams operating in an integrated health care approach*. One of the significant transitions affecting the AoD sector in Australia in recent years is the introduction of activity based funding approaches to non-admitted patient (community based) activity. Traditionally, these funding models reimburse services for ‘direct’ patient contacts – in which a clinician has face-to-face or telephone contact with the patient. Whilst this may be a suitable approach for many conventional AoD treatment models – such as counselling or withdrawal management, the experience of the pilot program highlighted the disproportionally high level of ‘indirect’ client activity (care co-ordination, team meetings, liaison between service providers) in the ACM services that are not reimbursed by activity based funding models. Further refinement of funding models for such integrated health care services are required, that recognise that the savings to the health system (e.g. fewer hospital presentations) are achieved by increased expenditure by another part of the health system – in this case community based ACM teams. In this regard, services such as ACM can be conceptualised as ‘hospital avoidance programs through integrated health models of care’, and further work is required to ensure such prevention services can be accommodated in sustainable funding models.

## Conclusion

In conclusion, an assertive care model targeting frequent ED attenders with severe SUDs appears to be a cost-effective approach to reducing preventable ED presentations and hospital inpatient admissions, particularly substance use related presentations. The pilot project demonstrated the cost-benefits to health services and improved patient outcomes, enabled the refinement of the model of care that better reflects patient needs, and served as the catalyst for the establishment of five AoD Assertive Community Management services across NSW. These services aim to provide an integrated health care approach for patients with severe SUDs and high utilisation of acute hospital services. Further evaluation of these expanded services is planned.

## Declarations

Ethics approval and consent to participate: The evaluation was approved by the SESLHD Human Research and Ethics Committee (no. LNR/14/POWH/483) and approval was granted for a waiver of consent for use and disclosure of medical information between the approved sites (SESLHD and St Vincents’ Hospital) for the purpose of the evaluation.

## Data Accessibility Statement

The dataset is not able to be made publically available as even with identifiers removed, data remain potentially re-identifiable. However, the authors will support access to the data on reasonable request, and will facilitate such an application to the reviewing human research ethics committee (South Eastern Sydney Local Health District Human Research Ethics Committee).

## Additional File

The additional file for this article can be found as follows:

10.5334/ijic.5343.s1 Appendix. Tables i (ED visit costs) and ii (Salary rates for IMPACT staff).

## Figures and Tables

**Table:** 

ACM	Assertive Community Management
ATOP	Australian Treatment Outcomes Profile
CGI-I	Clinical Global Impressions – Improvement
CL	Consultation/Liaison
D&A	Drug and Alcohol
ED/s	Emergency Department/s
IMPACT	**I**ntegrated **M**anagement **P**athways for **A**lcohol & drug **C**lients into **T**reatment
SESLHD	South Eastern Sydney Local Health District
SUD/s	Substance use disorder/s
