# Supplementary material for: Evaluation of an Assertive Management and Integrated Care Service for Frequent Emergency Department Attenders with Substance Use Disorders: The Impact Project: Evaluating an assertive management service for frequent ED attenders with substance use disorders
Source: Int J Integr Care. 2020 Apr 23;20(2):4. doi: 10.5334/ijic.5343 (PMC7181945; doi:10.5334/ijic.5343)
Supplement: Appendix. — Tables i (ED visit costs) and ii (Salary rates for IMPACT staff). [file ijic-20-2-5343-s1.pdf]

Appendix

Table i: Emergency department visit costs by triage code and admitted status, based on the Australian Public Hospitals Cost Report 2013-2014 Round 18 (16). Costs were adjusted to financial year 2015-2016 using Consumer Price Index change for health between December 2013 and December 2015 (10%), calculated using the mid-points of 2013-2014 and 2015-2016 financial years based on data released by the Australian Bureau of Statistics (17). Amounts are in Australian Dollars.

Admitted status	Triage code	Cost 2013-14	Cost 2015-16
Admitted	1	$1,815	$1,994.51
Admitted	2	$1,207	$1,326.37
Admitted	3	$942	$1,035.16
Admitted	4	$754	$828.57
Admitted	5	$594	$652.75
Deceased on arrival		$168	$184.62
Not admitted	1	$1,374	$1,509.89
Not admitted	2	$822	$903.30
Not admitted	3	$573	$629.67
Not admitted	4	$371	$407.69
Not admitted	5	$262	$287.91
Did not wait		$159	$174.73
Transfer presentation		$1,088	$1,195.60
Died in ED		$1,350	$1,483.52

Table ii: Salary rates used to calculate IMPACT staff costs, based on the NSW Health state award [22]. Amounts are in Australian Dollars.

**Profession**	**Calculation method**	**Hourly rate (salary only)**	**Hourly rate (with oncosts*)**
Social worker	Midpoint of level 2 year 4 and level 3 year 1 (midpoint of levels 1-6) – levels 7-8 not considered as they are managerial)	$44.15	$49.10
D&A specialist	Midpoint of levels 3 and 4 in NSW Health staff specialist award	$89.38	$98.63

*9.5% base salary as superannuation, plus $1,500 infrastructure support per worker per annum converted to an hourly rate based on a 38-hour week.
